# Correlation between Population Density and COVID-19 Cases during the Third Wave in Malaysia: Effect of the Delta Variant

**DOI:** 10.3390/ijerph19127439

**Published:** 2022-06-17

**Authors:** Nuur Hafizah Md Iderus, Sarbhan Singh Lakha Singh, Sumarni Mohd Ghazali, Cheong Yoon Ling, Tan Cia Vei, Ahmed Syahmi Syafiq Md Zamri, Nadhar Ahmad Jaafar, Qistina Ruslan, Nur Huda Ahmad Jaghfar, Balvinder Singh Gill

**Affiliations:** Institute for Medical Research, National Institutes of Health, Ministry of Health Malaysia, Shah Alam 40170, Selangor, Malaysia; lssarbhan@moh.gov.my (S.S.L.S.); sumarni.mg@moh.gov.my (S.M.G.); cheongyl@moh.gov.my (C.Y.L.); drciavei@moh.gov.my (T.C.V.); syahmi.syafiq@moh.gov.my (A.S.S.M.Z.); nadhar@moh.gov.my (N.A.J.); qistina@moh.gov.my (Q.R.); nurhuda.mj@moh.gov.my (N.H.A.J.); drbsgill@moh.gov.my (B.S.G.)

**Keywords:** COVID-19, population density, third wave, Delta variant

## Abstract

In this study, we describe the incidence and distribution of COVID-19 cases in Malaysia at district level and determine their correlation with absolute population and population density, before and during the period that the Delta variant was dominant in Malaysia. Methods: Data on the number of locally transmitted COVID-19 cases in each of the 145 districts in Malaysia, between 20 September 2020 and 19 September 2021, were manually extracted from official reports. The cumulative number of COVID-19 cases, population and population density of each district were described using choropleth maps. The correlation between population and population density with the cumulative number of COVID-19 cases in each district in the pre-Delta dominant period (20 September 2020–29 June 2021) and during the Delta dominant period (30 June 2021–19 September 2021) were determined using Pearson’s correlation. Results: COVID-19 cases were strongly correlated with both absolute population and population density (Pearson’s correlation coefficient (r) = 0.87 and r = 0.78, respectively). A majority of the districts had higher numbers of COVID-19 cases during the Delta dominant period compared to the pre-Delta period. The correlation coefficient in the pre-Delta dominant period was r = 0.79 vs. r = 0.86 during the Delta dominant period, whereas the pre-Delta dominant population density was r = 0.72, and in the Delta dominant period, r = 0.76. Conclusion: More populous and densely populated districts have a higher risk of transmission of COVID-19, especially with the Delta variant as the dominant circulating strain. Therefore, extra and more stringent control measures should be instituted in highly populated areas to control the spread of COVID-19.

## 1. Introduction

The World Health Organization (WHO) identified a new type of coronavirus, SARS-CoV-2, early in 2020, which causes the disease COVID-19. This novel coronavirus was first discovered in Wuhan, China, after its outbreak in December 2019. Following which, COVID-19 spread rapidly across the world in a short period of time, resulting in a Public Health Emergency of International Concern (PHEIC) [1]. As a result of this, COVID-19 was declared a pandemic by the WHO on 11 March 2020 [2]. The spread of the COVID-19 virus resulted in unprecedented outbreaks worldwide, characterized by exponential rises in new infections. As the pandemic progressed, many countries initially instituted several Public Health Social Measures (PHSM), followed by the more recent COVID-19 vaccination strategies to curb the COVID-19 outbreak. However, despite these measures, many countries are currently experiencing a resurgence in COVID-19 infections [3,4,5].

As of 28 February 2022, about 435 million COVID-19 infections and 5.95 million deaths due to COVID-19 have been reported globally, and more alarmingly, these estimates keep rising. The USA, India and Brazil are among those countries that have reported the highest numbers of COVID-19 infections globally. Moreover, in the South East Asia region, as of 28 February 2022, the incidence rates of COVID-19 infections were highest in Brunei Darussalam at 13,588.538 per 100,000 population, followed by Singapore (12,151.085 cases per 100,000 population) and Malaysia (10,565.52 cases per 100,000 population) [6]. In Malaysia, the first case of COVID-19 infection was reported on 22 January 2020, and this marked the beginning of the first wave with a total of 22 infections, which lasted until 26 February 2020. Following this, a much larger second wave began from 27 February 2020 to 19 September 2020, which resulted in 3375 infections. Currently, Malaysia is facing its third wave, which began on 20 September 2020 [7].

Since the beginning of the third wave in Malaysia, the distribution of COVID-19 infections across the country has varied, wherein several states, namely Selangor (30.2%), Johor (8.7%) and the Federal Territory of Kuala Lumpur (8.3%), have reported much larger numbers of COVID-19 infections compared to other states [8]. This observation could be attributed to the high population numbers and densities observed in these states, which could have increased the disease transmission. These findings were supported by evidence in the literature, which reports a higher distribution of COVID-19 cases in areas with larger densities [9], and significant correlations between population numbers and densities with COVID-19 cases in countries such as England, the USA and Turkey [10,11,12,13]. In addition to the above observations, a large number of COVID-19 infections were also observed in states with lesser densities, such as in Sarawak (9.3%) and Sabah (8.8%), during the third wave in Malaysia [8]. This is because despite these states having larger areas, which reduces the overall population density at the state level, they consist of multiple districts which are highly populous with large population densities. As a result of this, determining the effect of population and population density on COVID-19 cases at higher levels (i.e., state) may be inaccurate and misleading. Therefore, analysing this effect at lower levels (i.e., district level) would provide more meaningful and accurate findings.

To date, in Malaysia, there have been limited studies which examine the distribution of COVID-19 cases by districts and the relationship between absolute population and population density with COVID-19 cases during the third wave. This paper focuses specifically on the third wave of COVID-19, as there are several unique factors that affected the transmission dynamics of COVID-19 during the third wave, which differentiate it from the previous waves in Malaysia. These factors include, first, the presence of new and more virulent variants of the COVID-19 virus from 18 December 2020 onwards [14]. The emergence of these new variants posed an increased risk to the spread of the COVID-19 pandemic. Therefore, more measures were taken in the characterization of specific Variants of Interest (VOIs) and Variants of Concern (VOCs) to improve outbreak surveillance and control measures. For example, the Delta variant (B.1.617.2) designated as a VOC on 11 May 2021 was highly infectious and had affected many countries. This variant was found to be more transmissible and resulted in more severe forms of COVID-19 illness [14,15]. Malaysia recorded its first case of the Delta variant on 2 May 2021, which was detected in an Indian national screened at the Kuala Lumpur International Airport [16]. Subsequently, the first locally transmitted case of the Delta variant was detected on 17 May 2021 [17]. The presence of these VOCs, especially the Delta variant, intensified the outbreak during the third wave; therefore, when examining the relationship between absolute population and population density with COVID-19 cases during the third wave, it is important to account for the effects of the Delta variant.

The second factor unique to the third wave is the implementation of COVID-19 vaccination. Numerous studies have reported that vaccination had a major effect in decreasing COVID-19 infections [14,18]. The Malaysian vaccination program began on 24 February 2021 and was rolled out in phases. The first phase from February to April 2021, focused on frontliners, followed by the second phase involving senior citizens, high-risk groups and people with disabilities from April to August 2021, and from May 2021 onwards, for others aged 18 years and above. As of 19 September 2021, the last day of our study period, a total of 69.1% and 58% of the population had received one and two doses of the COVID-19 vaccine, respectively. The percentages of the total vaccine doses administered with the various vaccines are as follows, CoronaVac (inactivated SARS-CoV-2 vaccine by Sinovac) (46.5%) and Comirnaty (mRNA vaccine by Pfizer-BioNTech) (45.4%), Oxford AstraZeneca’s SARS-CoV-2 mRNA vaccine (7.8%) and less than 1% other vaccines. Malaysia’s vaccination program for those aged below 18 years was started on 20 September 2021, which was after our study period.

Due to the presence of these unique factors (i.e., VOCs and vaccination) during the third wave of COVID-19, it is important to determine the relationship between population and density with COVID-19 cases by accounting for demographic characteristics, vaccination status and the COVID-19 Delta variant, as it would provide a better understanding of the true unbiased relationship between population and density with COVID-19 cases. Therefore, the initial aims of this study are to describe the incidence and distribution of COVID-19 cases during the third wave in Malaysia. Subsequent to this, we determined the correlation between absolute population and population density with COVID-19 cases during the pre-Delta period, during Delta and in the overall period of the third wave. We believe the findings from this study would assist in the prioritization of instituting outbreak control measures based on disease distribution, to better control and manage the COVID-19 pandemic in Malaysia.

## 2. Materials and Methods

### 2.1. Data Source

Local COVID-19 case data were sourced from the Ministry of Health’s Malaysia official website (http://www.moh.gov.my), from 20 September 2020 to 19 September 2021. Local cases are defined as cases reported in 145 districts including three federal territories in Malaysia, based on the 2010 Malaysian census. An additional 13 districts which were formed after the 2010 Malaysian census were not included in the analysis, namely Pokok Sena, Bagan Datuk, Kalabakan, Telupid, Beluru, Bukit Mabong, Kabong, Pusa, Sebauh, Subis, Tanjung Manis, Tebedu and Telang Usan.

Imported cases were excluded in this study because the source of infection was outside of Malaysia and therefore did not contribute to local disease transmission. The population numbers and population density in each district were obtained from the Department of Statistics Malaysia (DOSM). The estimated population data were obtained from the DOSM, which is the authority that provides the official population statistics data for Malaysia. These yearly population estimates are generated by the DOSM using the cohort-component method, which is based on census data as well as rates of birth, death, and internal and international migration. In this study, the total populations used were projected 2020 mid-year populations, based on 2010 population census data. In addition, population density was defined as the district’s mid-year population for the year 2020 divided by its total land area (km^2^) [19]. Geospatial shape files were provided by the Department of Survey and Mapping Malaysia (JUPEM) in the year 2019.

### 2.2. Data Analysis

Data were analyzed using the Statistical Package for the Social Sciences (SPSS) version 26.0 release 2019 by International Business Machines, IBM Corp., Armonk, NY, USA [20]. Data were checked for missing data and abnormal values before performing any statistical analysis. There were no missing values. For the correlation analysis, the COVID-19 case data at district levels were categorized into three time periods, which were based on the detection of Delta variants in Malaysia. First was the pre-Delta variant period, which was from 20 September 2020 to 29 June 2021 (283 days). Second was the during-Delta variant period, which was from 30 June 2021 (the date the Delta variant became the predominantly circulating variant (more than 50%) among the samples tested by the Institute for Medical Research, Malaysia) to 19 September 2021 (82 days). The third time period was the one-year duration of the third wave, from 20 September 2020 to 19 September 2021 (365 days; the end date of 19 September 2021 was selected as it represented the downward trajectory of the third wave). The incidence of COVID-19 cases per 1000 populations by districts was estimated by dividing the total number of cases with the absolute population for each district. Quantum Geographic Information System (QGIS) version 3.10 was used to plot the incidence and distribution of COVID-19 cases by districts across total population and population densities.

Prior to the correlation analysis, the normality of absolute population, population density, and COVID-19 cases pre-Delta, during Delta and overall, were examined using the Shapiro–Wilk test and normal probability plots. The results of the Shapiro–Wilk test for all five variables were significant suggesting the data were not normally distributed and normal probability plots showed their deviations from the normal distribution (Appendix A). As the data were not normally distributed, log transformation was performed, and Pearson’s correlation coefficient (r) was used to determine the strength and direction of the correlation between absolute population and population density with COVID-19 cases. The classification of the strength of the relationship was determined based on the value of r which ranges from 0 to 1 where r = 0 indicates no association and r = −1 or +1 indicates perfect association with a *p*-value less than 0.05 indicating significant correlations. The magnitude of change for two variables is either in the same or in the opposite direction, indicated by a positive or negative value of the correlation coefficient [21]. Correlation analysis was conducted for all the three time periods to determine the effects of the Delta variant on the correlation between absolute population and population density with COVID-19 cases.

In addition, prior to the correlation analysis, multivariable linear regression analysis was performed with SPSS software to control for the confounding effects of sociodemographic factors (i.e., median household income and the percentage of the population aged 15 years old and above) and the percentage of the population fully vaccinated on the correlation between population density and COVID-19 cases [14,15]. All data were at district level, except for vaccination data which were available at state level only, and therefore, were used to represent each district’s vaccination percentage. Data were analyzed using a stepwise linear regression method. The cutoff probability for adding and removing variable in the stepwise method was 0.05 and 0.10 respectively. The final model was checked to ensure the assumptions of the analysis were sufficiently met [22].

## 3. Results

### 3.1. Characteristics of COVID-19 Cases in the Third Wave

The most populous and densely populated districts were Petaling, Selangor, (2,223,300 persons) and Kuala Lumpur (7863 people per square kilometer), respectively, in Malaysia (Appendix B).

**Figure 1:**
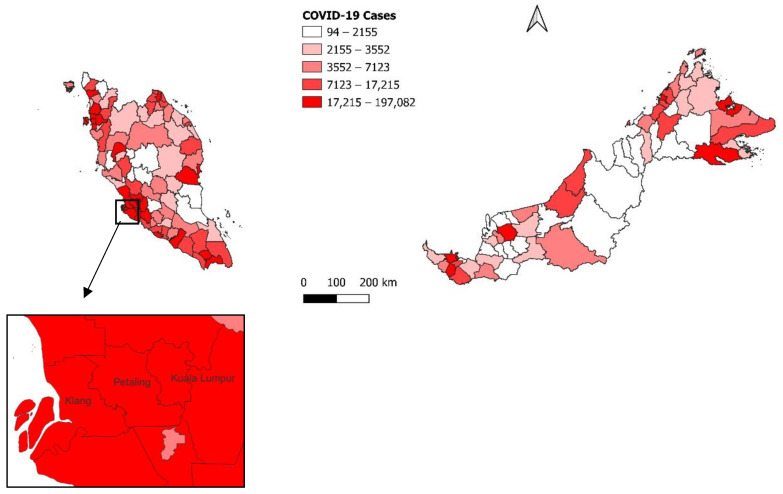
Distribution of COVID-19 cases by district, 20 September 2020 to 19 September 2021, Malaysia.

**Figure 2:**
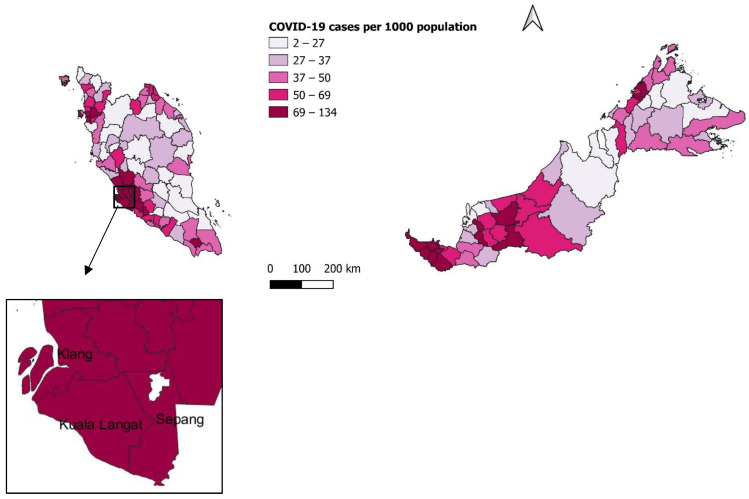
Distribution of COVID-19 incidence by district, 20 September 2020 to 19 September 2021, Malaysia.

For the overall time period, the highest number of cases were distributed in Petaling (*n* = 197,082 cases), followed by Kuala Lumpur (*n* = 178,406 cases) and Klang (*n* = 126,579 cases), as shown in Figure 1. The highest COVID-19 incidence rate was reported in Sepang (133.8 per 1000 population), followed by Klang (119.8 per 1000 population) and Kuala Langat (115.2 per 1000 population), as shown in Figure 2.

During the pre-Delta period, the highest number of cases was distributed in Kuala Lumpur (*n* = 73,041 cases), followed by Petaling (*n* = 72,839 cases) and Klang (*n* = 50,417 cases). In addition, the highest COVID-19 incidence rate was reported in Labuan (73.7 per 1000 population), followed by Sepang (62.8 per 1000 population) and Kapit (52.3 per 1000 population). During the Delta period, the highest number of cases was distributed in Petaling (*n* = 124,243 cases), followed by Kuala Lumpur (*n* = 105,365 cases) and Klang (*n* = 76,162 cases). The highest COVID-19 incidence rate was reported in Serian (87.5 per 1000 population), followed by Bau (79.0 per 1000 population) and Klang (72.1 per 1000 population) (Appendix C).

From the total of 145 districts, 70% (*n* = 127) of the districts reported an increase in both COVID-19 cases and the incidence rate during the Delta variant period compared to the pre-Delta period. The percentage increase in COVID-19 cases and the incidence rate ranged from 0.3% to 2500% and the mean increase was 242.5%. The COVID-19 cases and incidence rate per 1000 population for all the districts (*n* = 145) in the pre-Delta and during Delta periods in Malaysia are shown in Appendix C.

### 3.2. Association between Sociodemographic Factors and Vaccination with COVID-19 Cases

In multivariable regression analysis, population density and median household income were found to be independently associated with COVID-19 cases, after controlling for sociodemographic and vaccination factors. Following this analysis, population density alone accounted for 60% of the variation in COVID-19 cases. Moreover, 64% of the variation of the COVID-19 cases was explained by both population density and household income. This was a minimal increase of 4% contributed by the median household income variable, therefore suggesting household income does not largely affect COVID-19 cases (Table 1).

### 3.3. Correlation between Population and Population Density with COVID-19 Cases

**Figure 3:**
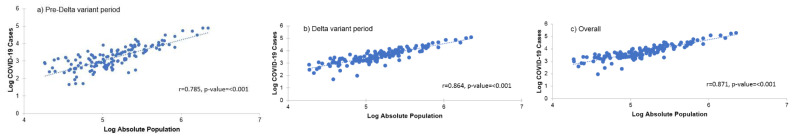
Correlation between absolute population and COVID-19 cases, (**a**) Pre-Delta variant period, (**b**) Delta variant period, (**c**) Overall period.

**Figure 4:**
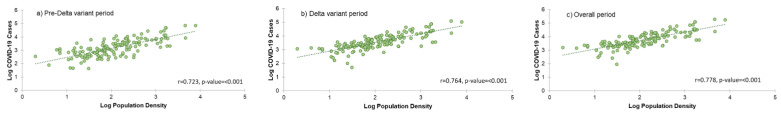
Correlation between population density and COVID-19 cases, (**a**) Pre-Delta variant period, (**b**) Delta variant period, (**c**) Overall period.

Correlation analysis showed both absolute population and population densities were significantly correlated (*p*-value < 0.001) with COVID-19 cases for the overall time period (r = 0.871), the pre-Delta variant period (r = 0.785) and the Delta variant period (r = 0.864), respectively, as shown in Figure 3. This corresponds to an increase in correlation of 15.9% from the pre-Delta period to the Delta period. The correlation between population density and COVID-19 cases was for the overall time period (r = 0.778), pre-Delta variant period (r = 0.723) and Delta variant period (r = 0.764) respectively as shown in Figure 4. This corresponds to an increase in correlation by 21.5% from the pre-Delta period to the Delta period.

Overall, an increase in the correlations between absolute population and population density with COVID-19 cases was observed during the Delta variant period compared to the pre-Delta period (Table 2). In addition, the correlation coefficient was higher for the correlations between absolute population and COVID-19 cases compared to the correlations between population density and COVID-19 cases, across all the time periods.

## 4. Discussion

In this study, we described the incidence and distribution of COVID-19 cases by districts as well as determining the correlations between absolute population and population density with COVID-19 cases during the third wave in Malaysia. In addition, the correlation findings of this study were analyzed and presented based on the pre-Delta variant period (20 September 2020 to 29 June 2021) and the Delta variant period (30 June 2021 to 19 September 2021) during the third wave.

The highest number of COVID-19 cases and incidence rate were observed in districts in Selangor state (i.e., Petaling and Klang) and the Federal Territory of Kuala Lumpur, during the third wave. This finding is observed primarily because these areas are highly urbanized, densely populated and populous. Similar findings have been reported in studies conducted in England and Malaysia [9,10]. In addition, this study also found high COVID-19 incidence in districts with lesser densities (i.e., Sepang). This finding could be attributed to the relatively small population numbers in these districts (i.e., Sepang = 265,600) and the higher number of COVID-19 infections (i.e., Sepang *n* = 35,539 cases), therefore ultimately resulting in higher incidence rates.

The findings of this study showed an increase in COVID-19 cases and the incidence rate between the pre-Delta and Delta variant periods, ranging from 0.3% to 2500%. Previous studies conducted in the countries which were affected by the Delta variant, such as England and United States, also reported a similar increase in COVID-19 cases [15,23,24]. The increment in COVID-19 cases and the incidence rate observed in this study is due to the effects of the Delta variant, and highly and densely populated areas which would increase disease transmission [15,23,24].

Furthermore, this study reports a positive significant correlation between absolute population and population densities with COVID-19 cases throughout the third wave. Our findings support the existing evidence that suggests COVID-19 cases tend to increase in areas that are highly and densely populated. Our findings were consistent with previous studies conducted in England, the United States, Turkey and Malaysia, which report higher COVID-19 cases and incidence in more densely populated areas [10,11,12]. Several reasons can be attributed to these findings. First, communities with high population numbers and population density have a higher probability of coming into contact with one another, therefore, increasing the risk of disease transmission [25,26,27,28]. In addition, individuals residing in densely populated areas tend to live in close proximities, which would result in prolonged, sustained and continuous exposure to possibly infected individuals, therefore, increasing disease transmission.

This study also reports an increase in the correlation between absolute population (15.9% correlation increase) and population densities (21.5% correlation increase) with COVID-19 cases, during the Delta period (16 May 2021 to 19 September 2021) compared to the pre-Delta period (20 September 2020 to 15 May 2021). The mild increase in the magnitude of the correlation across these two periods could be attributed to the fact that population density itself fuels COVID-19 disease transmission (resulting in high pre-Delta correlation estimates). In addition, the modest increase in the correlation supports the low transmissibility of the Delta variant. The relevance of this finding suggests that in highly and densely populated areas, the existence of a variant of concern with low disease transmissibility would contribute to an increase in the number of infections [15,18,29].

While many countries are still working on finding curative treatments and increasing COVID-19 immunization rates, non-pharmaceutical interventions (NPI) are still important measures to control and manage this pandemic. With limited resources and the need for timely institutions of NPI measures, many countries have adopted targeted outbreak control measures [10,27,30,31]. The findings from this study highlight the importance of implementing NPI in areas that are highly and densely populated as a priority, in order to control and manage the COVID-19 outbreak effectively. Moreover, the evidence generated from this study could be used to guide decision makers in making sound decisions regarding instituting targeted outbreak control measures. In addition, this study also provides evidence on the effects of a variant of concern (i.e., the Delta variant) on the correlation between absolute population and population density with COVID-19 cases, wherein such VOCs could be the driving factor in increasing disease transmissibility, especially in areas that are highly and densely populous.

To the best of our knowledge, this is the first study that has analyzed the correlation between absolute population and population density with COVID-19 cases, at different time frames in relation to the Delta variant during the third wave, to describe the changes in incidence rates and correlations caused by the Delta variant. This study has several strengths, which include first using districts as the smallest point for correlation analysis. By doing so, we were able to examine this correlation in more focused smaller areas, which would improve the precision and accuracy of the correlation instead of using larger areas such as states. Second, a longer study period (365 days) was used to examine the correlation, therefore improving the analysis and suggesting that the current data used are sufficient to show the impact of absolute population and population density on COVID-19 cases. Third, this study focused on local cases during the third wave of COVID-19, which contributed more than 90% of the total COVID-19 cases in Malaysia. Finally, this study ruled out the presence of potential confounders (i.e., household income, population aged 15 years and above and vaccination coverage) prior to examining the correlation between population density and COVID-19 cases.

The limitations of this study include the distribution of COVID-19 cases which may depend on a variety of other factors, which include geographical characteristics, economic growth, health infrastructure, regulatory policy and the number of tests. In addition, it would also be important to examine the correlation and relationship of other sociodemographic and socioeconomic factors with COVID-19 mortality in Malaysia. Therefore, further research may be needed to account for the aforementioned issues.

## 5. Conclusions

In conclusion, the present study reports that a higher incidence of COVID-19 infections was found among highly and densely populated districts, especially during the Delta variant period in the third wave in Malaysia. In addition, absolute population and population density significantly contribute to the increase in COVID-19 infections, as evident from the positive correlations reported in this study. Therefore, prioritizing the implementation of outbreak control measures in highly and densely populated areas and with the presence of VOCs could be key to containing this highly infectious disease and eventually controlling the COVID-19 pandemic.

## Figures and Tables

**Table 1:** Multivariable analysis between COVID-19 cases and sociodemographic factors.

Factor	Crude Coefficients, B (95% CI)	*p*-Value	Adjusted Coefficients, B (95% CI)	Std. Error	*p*-Value
Age 15 and above	1222.87 (223.87, 2221.88)	0.017			
Vaccination	674.60 (371.644, 977.555)	<0.001			
Median household income (RM)	11.58 (9.360, 13.788)	<0.001	4.88 (2.64, 7.114)	1.132	<0.001
Population density	23.28 (20.152, 26.409)	<0.001	18.10 (14.305, 21.887)	1.918	<0.001

**Table 2:** Correlation analysis in relation to Delta variant during the third wave in Malaysia, 20 September 2020 to 19 September 2021.

	Absolute Population and COVID-19 Cases		Population Density and COVID-19 Cases	
	Correlation (r)	*p*-Value	Correlation (r)	*p*-Value
Pre-Delta variant period	0.785	<0.001 *	0.723	<0.001 *
Delta variant period	0.864	<0.001 *	0.764	<0.001 *
Overall period	0.871	<0.001 *	0.778	<0.001 *

Note. * Significance set at *p* < 0.05.

**Figure A1:**
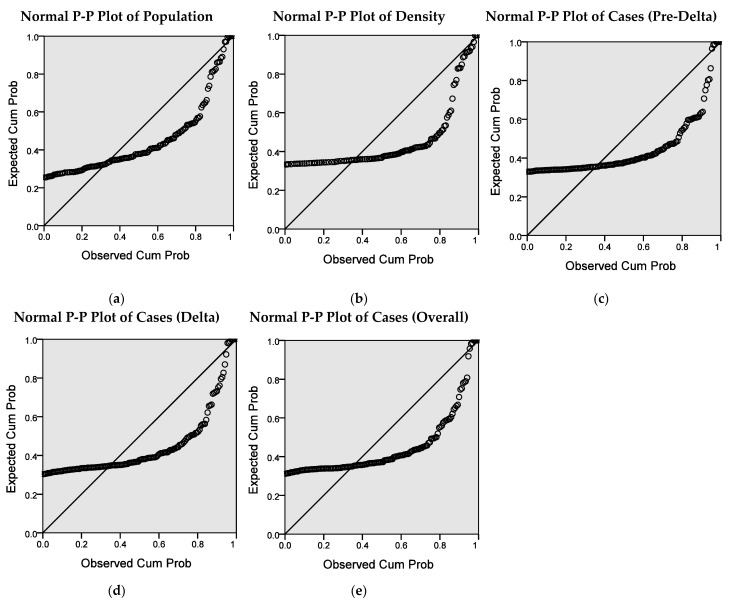
Normal P–P Plot, (**a**) Population, (**b**) Density, (**c**) COVID-19 cases during pre-Delta variant, (**d**) Delta variant period, (**e**) Overall period.

**Figure A2:**
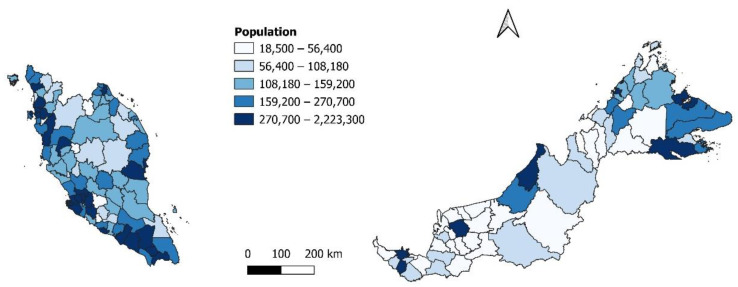
Absolute population by district, Malaysia (projected year 2020).

**Figure A3:**
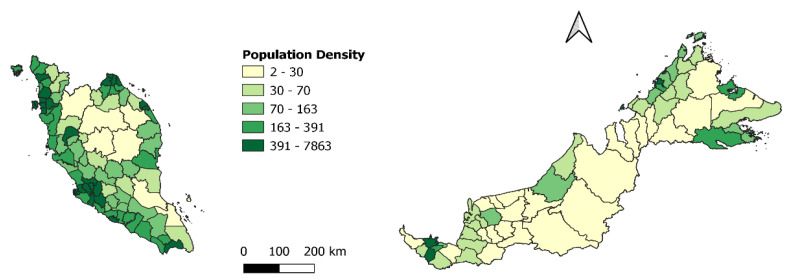
Population density by district, Malaysia (projected year 2020).

## Data Availability

The datasets used and analyzed during the current study are available from the Ministry of Health Malaysia website and provided by the Department of Statistics Malaysia.

## Appendix A

**Table A1:** Results of Shapiro–Wilk Test for normality of population, density and COVID-19 cases pre-Delta, during Delta and overall.

	Shapiro–Wilk, W	df	*p*-Value
Population	0.570	145	<0.001
Density	0.427	145	<0.001
COVID-19 cases pre-Delta variant	0.422	145	<0.001
COVID-19 cases during Delta variant	0.471	145	<0.001
Overall COVID-19 cases	0.447	145	<0.001

## Appendix C

**Table A2:** Incidence rate of COVID-19 per 1000 population by districts, 20 September 2020 to 19 September 2021.

No	State	District	Population	Population Density (Person per sqft/km)	COVID-19 Cases			Incidence per 1000 Population		
					Pre-Delta Variant Period	Delta Variant Period	Overall	Pre-Delta Variant Period	Delta Variant Period	Overall Study Period
1	Johor	Batu Pahat	488,800	249	4997	8572	13,569	10.2	17.5	27.8
2		Johor Bahru	1,621,400	1521	29,201	45,386	74,587	18.0	28.0	46.0
3		Kluang	351,700	123	3258	9901	13,159	9.3	28.2	37.4
4		Kota Tinggi	231,300	66	4062	5798	9860	17.6	25.1	42.6
5		Kulai	294,800	390	8506	12,526	21,032	28.9	42.5	71.3
6		Mersing	85,100	30	501	1701	2202	5.9	20.0	25.9
7		Muar	288,900	207	7783	9658	17,441	26.9	33.4	60.4
8		Pontian	183,100	196	3070	5051	8121	16.8	27.6	44.4
9		Segamat	221,600	77	1768	3978	5746	8.0	18.0	25.9
10		Tangkak	159,800	164	2355	4088	6443	14.7	25.6	40.3
11	Kedah	Baling	158,700	104	968	8225	9193	6.1	51.8	57.9
12		Bandar Baharu	49,300	182	521	2314	2835	10.6	46.9	57.5
13		Kota Setar	423,400	1008	6477	15,779	22,256	15.3	37.3	52.6
14		Kuala Muda	527,900	578	7700	28,286	35,986	14.6	53.6	68.2
15		Kubang Pasu	257,800	273	1901	5523	7424	7.4	21.4	28.8
16		Kulim	334,100	432	2657	23,071	25,728	8.0	69.1	77.0
17		Langkawi	113,100	215	325	4307	4632	2.9	38.1	41.0
18		Padang Terap	74,100	55	275	1556	1831	3.7	21.0	24.7
19		Pendang	111,600	177	495	4034	4529	4.4	36.1	40.6
20		Sik	79,400	49	218	1999	2217	2.7	25.2	27.9
21		Yan	80,000	325	443	1736	2179	5.5	21.7	27.2
22	Kelantan	Bachok	169,100	607	2417	7008	9425	14.3	41.4	55.7
23		Gua Musang	118,700	15	554	3690	4244	4.7	31.1	35.8
24		Jeli	53,000	40	701	1970	2671	13.2	37.2	50.4
25		Kota Bharu	620,500	1541	13,993	21,099	35,092	22.6	34.0	56.6
26		Kuala Krai	140,500	62	967	2545	3512	6.9	18.1	25.0
27		Machang	118,200	225	1696	2496	4192	14.3	21.1	35.5
28		Pasir Mas	241,100	423	4442	7155	11,597	18.4	29.7	48.1
29		Pasir Puteh	148,900	352	1889	6901	8790	12.7	46.3	59.0
30		Tanah Merah	155,100	176	2437	5247	7684	15.7	33.8	49.5
31		Tumpat	194,700	1083	3273	7468	10,741	16.8	38.4	55.2
32	Melaka	Alor Gajah	215,100	319	4100	9164	13,264	19.1	42.6	61.7
33		Jasin	158,800	234	4103	5237	9340	25.8	33.0	58.8
34		Melaka Tengah	586,600	1634	8299	20,605	28,904	14.1	35.1	49.3
35	Negeri Sembilan	Jelebu	45,700	34	1083	756	1839	23.7	16.5	40.2
36		Jempol	133,200	90	1249	3003	4252	9.4	22.5	31.9
37		Kuala Pilah	75,200	73	1949	2227	4176	25.9	29.6	55.5
38		Port Dickson	131,800	226	4032	5072	9104	30.6	38.5	69.1
39		Rembau	49,400	122	2142	2498	4640	43.4	50.6	93.9
40		Seremban	631,000	662	28,345	33,309	61,654	44.9	52.8	97.7
41		Tampin	96,400	113	754	1647	2401	7.8	17.1	24.9
42	Pahang	Bentong	136,800	75	2106	4651	6757	15.4	34.0	49.4
43		Bera	113,500	51	405	1770	2175	3.6	15.6	19.2
44		Cameron Highlands	44,100	62	215	1017	1232	4.9	23.1	27.9
45		Jerantut	106,800	14	951	1979	2930	8.9	18.5	27.4
46		Kuantan	536,800	181	4367	19,542	23,909	8.1	36.4	44.5
47		Lipis	105,300	20	429	1301	1730	4.1	12.4	16.4
48		Maran	136,600	71	560	1633	2193	4.1	12.0	16.1
49		Pekan	131,300	35	398	1544	1942	3.0	11.8	14.8
50		Raub	109,100	48	534	810	1344	4.9	7.4	12.3
51		Rompin	137,100	26	329	1341	1670	2.4	9.8	12.2
52		Temerloh	192,400	85	1299	5790	7089	6.8	30.1	36.8
53	Perak	Batang Padang	131,900	73	807	6368	7175	6.1	48.3	54.4
54		Hilir Perak	157,700	199	3835	2990	6825	24.3	19.0	43.3
55		Hulu Perak	105,200	16	751	1541	2292	7.1	14.6	21.8
56		Kampar	109,400	163	365	2300	2665	3.3	21.0	24.4
57		Kerian	199,300	221	1112	4153	5265	5.6	20.8	26.4
58		Kinta	841,700	645	5926	19,322	25,248	7.0	23.0	30.0
59		Kuala Kangsar	177,900	70	730	3701	4431	4.1	20.8	24.9
60		Larut Matang & Selama	367,900	180	3780	11,225	15,005	10.3	30.5	40.8
61		Manjung	258,400	221	3022	3983	7005	11.7	15.4	27.1
62		Muallim	73,200	78	474	1664	2138	6.5	22.7	29.2
63		Perak Tengah	114,300	89	340	1340	1680	3.0	11.7	14.7
64	Perlis	Kangar	264,700	323	286	1803	2089	1.1	6.8	7.9
65	Pulau Pinang	Barat Daya	237,000	1356	7463	11,507	18,970	31.5	48.6	80.0
66		Seberang Perai Selatan	198,000	817	6052	14,223	20,275	30.6	71.8	102.4
67		Seberang Perai Tengah	438,400	1844	7503	25,119	32,622	17.1	57.3	74.4
68		Seberang Perai Utara	344,900	1288	4198	16,125	20,323	12.2	46.8	58.9
69		Timur Laut	588,200	4653	8645	15,933	24,578	14.7	27.1	41.8
70	Sabah	Beaufort	85,100	49	754	3098	3852	8.9	36.4	45.3
71		Beluran	135,300	25	285	1932	2217	2.1	14.3	16.4
72		Keningau	222,700	63	2391	5170	7561	10.7	23.2	34.0
73		Kinabatangan	200,600	30	1279	3079	4358	6.4	15.3	21.7
74		Kota Belud	113,700	82	1576	3802	5378	13.9	33.4	47.3
75		Kota Kinabalu	581,700	1659	14,174	23,651	37,825	24.4	40.7	65.0
76		Kota Marudu	82,600	43	627	2882	3509	7.6	34.9	42.5
77		Kuala Penyu	25,000	55	235	443	678	9.4	17.7	27.1
78		Kudat	103,200	79	2016	2096	4112	19.5	20.3	39.8
79		Kunak	81,500	72	1538	1243	2781	18.9	15.3	34.1
80		Lahad Datu	263,100	35	6295	4081	10,376	23.9	15.5	39.4
81		Nabawan	40,600	7	613	1091	1704	15.1	26.9	42.0
82		Papar	171,000	135	2478	6085	8563	14.5	35.6	50.1
83		Penampang	155,300	366	3706	9571	13,277	23.9	61.6	85.5
84		Pitas	46,000	32	349	1810	2159	7.6	39.3	46.9
85		Putatan	73,100	1808	2154	3461	5615	29.5	47.3	76.8
86		Ranau	115,900	32	532	2259	2791	4.6	19.5	24.1
87		Sandakan	518,200	229	7511	10,324	17,835	14.5	19.9	34.4
88		Semporna	175,800	154	2656	653	3309	15.1	3.7	18.8
89		Sipitang	46,000	17	359	2301	2660	7.8	50.0	57.8
90		Tambunan	44,100	32	343	630	973	7.8	14.3	22.1
91		Tawau	521,000	233	10,690	9118	19,808	20.5	17.5	38.0
92		Tenom	70,000	29	245	1658	1903	3.5	23.7	27.2
93		Tongod	44,900	4	81	1355	1436	1.8	30.2	32.0
94		Tuaran	130,500	110	3176	8660	11,836	24.3	66.4	90.7
95	Sarawak	Asajaya	37,900	125	272	1703	1975	7.2	44.9	52.1
96		Bau	62,200	70	556	4914	5470	8.9	79.0	87.9
97		Belaga	44,500	2	357	1182	1539	8.0	26.6	34.6
98		Betong	73,600	48	792	2129	2921	10.8	28.9	39.7
99		Bintulu	229,300	115	7850	5867	13,717	34.2	25.6	59.8
100		Dalat	23,300	26	391	337	728	16.8	14.5	31.2
101		Daro	37,900	31	43	51	94	1.1	1.3	2.5
102		Julau	18,700	11	691	302	993	37.0	16.1	53.1
103		Kanowit	34,300	15	1526	590	2116	44.5	17.2	61.7
104		Kapit	65,800	17	3440	1067	4507	52.3	16.2	68.5
105		Kuching	711,500	475	7915	44,407	52,322	11.1	62.4	73.5
106		Lawas	46,200	12	48	258	306	1.0	5.6	6.6
107		Limbang	56,900	14	47	600	647	0.8	10.5	11.4
108		Lubok Antu	33,100	11	207	794	1001	6.3	24.0	30.2
109		Lundu	39,200	22	326	2825	3151	8.3	72.1	80.4
110		Maradong	34,800	48	1422	2386	3808	40.9	68.6	109.4
111		Marudi	76,900	25	158	101	259	2.1	1.3	3.4
112		Matu	21,400	13	233	166	399	10.9	7.8	18.6
113		Miri	356,900	69	6954	2883	9837	19.5	8.1	27.6
114		Mukah	52,300	21	1656	1923	3579	31.7	36.8	68.4
115		Pakan	18,500	20	759	761	1520	41.0	41.1	82.2
116		Samarahan	102,700	252	1185	6443	7628	11.5	62.7	74.3
117		Saratok	54,400	61	376	1504	1880	6.9	27.6	34.6
118		Sarikei	67,400	68	1319	999	2318	19.6	14.8	34.4
119		Selangau	27,400	7	1258	933	2191	45.9	34.1	80.0
120		Serian	105,800	60	999	9253	10,252	9.4	87.5	96.9
121		Sibu	288,000	129	12,107	7796	19,903	42.0	27.1	69.1
122		Simunjan	46,900	21	112	2341	2453	2.4	49.9	52.3
123		Song	24,500	6	1089	1216	2305	44.4	49.6	94.1
124		Sri Aman	78,300	34	1446	2212	3658	18.5	28.3	46.7
125		Tatau	36,900	7	947	1150	2097	25.7	31.2	56.8
126	Selangor	Gombak	842,200	1290	24,411	47,832	72,243	29.0	56.8	85.8
127		Hulu Langat	1,413,100	1697	47,840	75,478	123,318	33.9	53.4	87.3
128		Hulu Selangor	245,700	140	5566	11,592	17,158	22.7	47.2	69.8
129		Klang	1,056,200	1672	50,417	76,162	126,579	47.7	72.1	119.8
130		Kuala Langat	279,100	326	13,069	19,078	32,147	46.8	68.4	115.2
131		Kuala Selangor	259,900	219	7791	11,624	19,415	30.0	44.7	74.7
132		Petaling	2,223,300	4565	72,839	124,243	197,082	32.8	55.9	88.6
133		Sabak Bernam	130,500	130	1285	2204	3489	9.8	16.9	26.7
134		Sepang	265,600	482	16,667	18,872	35,539	62.8	71.1	133.8
135	Terengganu	Besut	175,800	143	2745	3910	6655	15.6	22.2	37.9
136		Dungun	193,300	71	1049	6157	7206	5.4	31.9	37.3
137		Hulu Terengganu	90,700	23	807	1550	2357	8.9	17.1	26.0
138		Kemaman	216,100	85	202	5252	5454	0.9	24.3	25.2
139		Kuala Nerus	157,900	397	928	3676	4604	5.9	23.3	29.2
140		Kuala Terengganu	268,600	1278	1698	8905	10,603	6.3	33.2	39.5
141		Marang	121,700	183	629	2804	3433	5.2	23.0	28.2
142		Setiu	69,900	54	1060	2245	3305	15.2	32.1	47.3
143	Federal Territory	Kuala Lumpur	1,910,700	7863	73,041	105,365	178,406	38.2	55.1	93.4
144		Putrajaya	94,600	2090	2147	3572	5719	22.7	37.8	60.5
145		Labuan	103,100	1028	7601	1856	9457	73.7	18.0	91.7

Note: Pre-Delta variant period = 20 September 2020–15 May 2021; Delta variant period = 16 May 2021–19 September 2021; Overall study period = 20 September 2020–19 September 2021.
